# Diagnostic performance of 3D TSE MRI versus 2D TSE MRI of the knee at 1.5 T, with prompt arthroscopic correlation, in the detection of meniscal and cruciate ligament tears*

**DOI:** 10.1590/0100-3984.2015.0042

**Published:** 2016

**Authors:** Francisco Abaeté Chagas-Neto, Marcello Henrique Nogueira-Barbosa, Mário Müller Lorenzato, Rodrigo Salim, Maurício Kfuri-Junior, Michel Daoud Crema

**Affiliations:** 1 PhD, Professor of Radiology, Division of Radiology, Universidade de Fortaleza (Unifor) and Centro Universitário Christus; Section Head, Musculoskeletal Imaging, Hospital Antonio Prudente, Fortaleza, CE, Brazil.; 2 PhD, Full Professor of Radiology, Division of Radiology, Internal Medicine Department, Faculdade de Medicina de Ribeirão Preto da Universidade de São Paulo (FMRP-USP), Ribeirão Preto, SP, Brazil.; 3 MD, Radiologist, Radiologia Especializada de Ribeirão Preto, Ribeirão Preto, SP, Brazil.; 4 PhD, Department of Biomechanics, Medicine and Rehabilitation of the Locomotor Apparatus, Faculdade de Medicina de Ribeirão Preto da Universidade de São Paulo (FMRP-USP), Ribeirão Preto, SP, Brazil.; 5 MD, Radiologist, Radiology Department, Saint-Antoine Hospital, University of Paris VI, Paris, France; Department of Radiology, Quantitative Imaging Center, Boston University School of Medicine, Boston, MA, USA; Department of Radiology and Teleimagem, Hospital do Coração (HCor), São Paulo, SP, Brazil.

**Keywords:** Knee, Menisci, Anterior cruciate ligament, Magnetic resonance imaging, Arthroscopy

## Abstract

**Objective:**

To compare the diagnostic performance of the three-dimensional turbo spin-echo (3D TSE) magnetic resonance imaging (MRI) technique with the performance of the standard two-dimensional turbo spin-echo (2D TSE) protocol at 1.5 T, in the detection of meniscal and ligament tears.

**Materials and Methods:**

Thirty-eight patients were imaged twice, first with a standard multiplanar 2D TSE MR technique, and then with a 3D TSE technique, both in the same 1.5 T MRI scanner. The patients underwent knee arthroscopy within the first three days after the MRI. Using arthroscopy as the reference standard, we determined the diagnostic performance and agreement.

**Results:**

For detecting anterior cruciate ligament tears, the 3D TSE and routine 2D TSE techniques showed similar values for sensitivity (93% and 93%, respectively) and specificity (80% and 85%, respectively). For detecting medial meniscal tears, the two techniques also had similar sensitivity (85% and 83%, respectively) and specificity (68% and 71%, respectively). In addition, for detecting lateral meniscal tears, the two techniques had similar sensitivity (58% and 54%, respectively) and specificity (82% and 92%, respectively). There was a substantial to almost perfect intraobserver and interobserver agreement when comparing the readings for both techniques.

**Conclusion:**

The 3D TSE technique has a diagnostic performance similar to that of the routine 2D TSE protocol for detecting meniscal and anterior cruciate ligament tears at 1.5 T, with the advantage of faster acquisition.

## Abstract (pt)

**Objetivo:**

Comparar o desempenho diagnóstico da técnica tridimensional turbo spin-eco (3D TSE) de ressonância magnética (RM) do joelho na detecção de rupturas meniscais e ligamentares em comparação com o protocolo bidimensional turbo spin-eco (2D TSE).

**Materiais e Métodos:**

A sequência 3D TSE foi adicionada ao protocolo de rotina 2D TSE em 38 pacientes que foram submetidos a cirurgia artroscópica do joelho em até três dias após a realização da RM. Usando os achados artroscópicos como referência padrão ouro, foram calculados o desempenho diagnóstico e a concordância entre os protocolos.

**Resultados:**

A técnica 3D TSE e o protocolo 2D TSE apresentaram, respectivamente, sensibilidade (93%/93%) e especificidade (80%/ 85%) semelhantes na detecção de rupturas do ligamento cruzado anterior, sensibilidade (85%/83%) e especificidade (68%/71%) semelhantes na detecção de rupturas do menisco medial, assim como sensibilidade (58%/54%) e especificidade (82%/92%) semelhantes na detecção de rupturas do menisco lateral. A concordância intraobservador entre os dois métodos foi de substancial a quase perfeita em todos os parâmetros avaliados para ambos os leitores.

**Conclusão:**

A técnica 3D- SE apresentou desempenho diagnóstico semelhante ao protocolo de rotina 2D TSE na detecção de rupturas meniscais e ligamentares em magneto de 1,5 T, com a vantagem de possibilitar uma redução significativa no tempo de aquisição.

## INTRODUCTION

Routine magnetic resonance imaging (MRI) protocols for the knee often use fluid-sensitive sequences based on two-dimensional (2D) acquisitions, acquired in three orthogonal planes (coronal, axial, and sagittal). These sequences are widely used in clinical practice as well as in clinical research^(1-3)^.

Although 2D sequences exhibit high spatial resolution, they are acquired with relatively thick slices (2.0-4.0 mm) and may present gaps between slices, which can generate partial volume artifacts. When 2D sequences are the source for reformation, it is also impossible to generate reconstructions in multiple planes without a significant loss of quality.

Three-dimensional (3D) turbo spin-echo (TSE) MRI with isotropic or nearly isotropic resolution techniques has the potential to improve the depiction of pathology by reducing partial volume averaging as well as time efficiency of the MRI use in the musculoskeletal system^(1,4-7)^. Such volumetric acquisitions may be used in order to create multiplanar reconstructions, thereby eliminating the need to repeat sequences, providing spin-echo tissue contrast in different planes. In addition, the acquisition of source images is faster with 3D TSE than with the triplanar 2D TSE protocol^(1,4-7)^.

If the 3D technique can provide at least the same quality of assessment as does the 2D technique and do it in less time, with the possibility of multiplanar reconstruction, the differences between 2D and 3D TSE could have a significant effect on clinical practice and research. The majority of studies assessing the diagnostic performance of 3D TSE MRI in detecting pathology of the knee have used 3.0 T scanners^(1,4-10)^, and there are therefore few data available regarding the application of the technique in the more widely available 1.5 T scanners. There are, however, some controversies and disadvantages that should be mentioned before one considers replacing the routine 2D TSE protocol with the 3D TSE technique in the assessment of the knee. Acquisition times are typically longer for the 3D technique than for the single-acquisition 2D technique, which makes the former more susceptible to motion artifacts. Decreasing the time of acquisition for 3D techniques, preferable for a busy clinical practice, is often challenging and will likely have an impact on the quality of the images acquired. Recent studies have demonstrated that routine 2D protocols are more reliable in depicting meniscal pathology than is the 3D protocol, that difference likely being related to decreased in-plane resolution and image blurring^(10)^, as well as to a decrease in the number of signals averaged (to accelerate the acquisition time) and to suboptimal soft-tissue contrast^(8)^.

The main purpose of this study was to evaluate the diagnostic performance of 3D TSE MRI at 1.5 T in the detection of meniscal and ligament tears, in comparison with that of the standard 2D TSE protocol, using prompt surgical findings (arthroscopy) as the reference standard. Our hypothesis was that a single acquisition with the 3D TSE technique with multiplanar reconstructions would have a diagnostic performance similar to that of the 2D TSE routine protocol, at 1.5 T.

## MATERIALS AND METHOD

### Participants

After approval by the local institutional review board, we recruited participants who agreed to take part after explanation of research methods and objectives. All participants gave written informed consent. We evaluated 38 consecutive patients who had been referred for knee arthroscopy and agreed to participate in this prospective study by having an MRI of the knee prior to surgery. There were three main indications for the patients scheduled for surgery and included in this study: knee instability, joint locking, and chronic joint pain without improvement after clinical management. The study sample comprised 28 men and 10 women, from 21 to 57 years of age (mean, 33.5 ± 10.4 years).

### MRI acquisition

All knees were imaged with the same 1.5 T MRI scanner (Philips Achieva 1.5 T MRI System; Philips Medical Systems, Best, The Netherlands) and an 8-channel SENSE knee coil. Routine 2D and 3D TSE images were acquired on the same day. The MRI parameters and acquisition time for both techniques are summarized in Table 1.

**Table 1:** MRI parameters for the 2D and 3D techniques. All sequences were acquired with intermediate-weighted spectral adiabatic inversion recovery.

MRI parameter	Sagittal 2D TSE	Coronal 2D TSE	Axial 2D TSE	Sagittal source 3D TSE
Repetition time (ms)	2342	2342	3045	2500
Echo time (ms)	50	50	50	35
Matrix (pixels)	224 × 176	224 × 176	224 × 176	300 × 258
Field of view (cm)	16	16	16	18
Slice thickness (mm)	4	4	4	0.6 × 0.6 × 0.7
Echo train (n)	14	14	14	65
Excitations (n)	4	4	4	1
Bandwidth (kHz)	395	386	429	255
Acquisition time	2 min 43 sec*	2 min 30 sec*	3 min*	5 min^†^

* Total 2D TSE multiplanar acquisition time: 8 minutes 13 seconds.

† Total 3D TSE single sagittal plane acquisition time: 5 minutes (approximately 40% less than in

The sagittal source images from the 3D TSE technique were used in order to create sagittal, coronal, and axial reformatted images of the knee joint with a slice thickness of 1.5 mm. The reformatted images were used for the 3D TSE assessment of the knee.

The post-processing of the 3D TSE sequence was performed by a fellow in musculoskeletal radiology on a Philips Achieva MRI workstation (Extended MR Workspace; Philips Medical Systems) immediately after the images had been acquired.

### MRI assessment

Images were interpreted independently by two musculoskeletal radiologists. Reader 1 was a senior radiologist with ten years of experience, and reader 2 was a fellow in musculoskeletal radiology with one year of training. Both were blinded to all clinical information about the MRI. The 2D and 3D TSE images were assessed separately and independently by both radiologists. They assessed the 3D images first and the 2D images after a minimum interval of four weeks. The delay in the second reading was intended to minimize the risk of interpretation and recognition bias.

The medial and lateral menisci were evaluated throughout their length (anterior horn, body, and posterior horn) and were classified according to the presence or absence of a meniscal tear. A meniscal tear was defined as either meniscal distortion or intermediate to high signal intensity extending into the articular surface of the meniscus on at least two consecutive sagittal or coronal images^(11)^. Intrameniscal signal changes were not considered indicative of meniscal tears.

The anterior cruciate ligament (ACL) and posterior cruciate ligament (PCL) were both analyzed according to the presence or absence of tears, regardless of whether the tears were partial or complete. The signs of cruciate ligament tear were an abnormal course, abnormal signal intensity, and partial or complete discontinuity^(12-14)^.

### Arthroscopic knee surgery

All knee arthroscopies were performed within three days after the MRI, 80% being performed on the same day. The arthroscopies were performed by one of two experienced knee surgeons at our institution with 5 and 20 years of practice, respectively. During all arthroscopies, a complete inventory of the joint was performed, with special attention to and documentation of menisci and cruciate ligaments, which were assessed according to the presence or absence of tears.

### Analytic approach

The sensitivity, specificity, positive predictive value, negative predictive value, and accuracy for the detection of meniscal and cruciate ligament tears were calculated separately for each of the MRI protocols, using surgical findings (arthroscopy) as the reference standard. To increase the statistical power for the comparison between the two imaging protocols^(4)^, the data from the independent reviews of both readers were assessed separately and then mathematically combined when calculating sensitivity, specificity, positive predictive value, negative predictive value and accuracy. Kappa statistics were used in order to measure the interobserver agreement, as well as to determine the intraobserver agreement between the two methods, for each reader^(15)^.

To calculate the differences in diagnostic performance between the 3D TSE and the 2D TSE MRI techniques for dichotomized values (presence or absence of pathology), we used Fisher's exact test, with 95% confidence intervals, values of *p* < 0.05 being considered statistically significant. The statistical analysis was performed with the Statistical Package for the Social Sciences, version 17.0.2 (SPSS Inc., Chicago, IL, USA).

## RESULTS

The number of positive findings (tears) detected by each reader in each MRI technique is presented in Table 2, as is the number of positive findings detected by arthroscopy. The overall combined sensitivity, specificity, and accuracy for the detection of tears of the ACL, the medial meniscus, and the lateral meniscus are displayed in Table 3.

**Table 2:** Number of positive findings detected by each reader in each MRI technique assessed.

Reader	Technique	Medial meniscus	Lateral meniscus	ACL tears
		tears (*n* = 24)	tears (*n* = 13)	(*n* = 28)
1	2D MRI	20	7	26
1	3D MRI	20	7	27
2	2D MRI	20	7	26
2	3D MRI	21	8	25

*n*, number of positive findings at arthroscopy.

**Table 3:** Overall combined sensitivity, specificity, accuracy, and agreement (kappa) for 2D and 3D MRI protocols in the detection of tears of the anterior cruciate ligament, medial meniscus, and lateral meniscus.

	2D: both readers combined						3D: both readers combined						
	Sensitivity		Specificity		Accuracy		Sensitivity		Specificity		Accuracy		Overall 2D vs. 3D agreement
MRI feature	% [95% CI]		% [95% CI]		% [95% CI]		% [95% CI]		% [95% CI]		% [95% CI]		Kappa [95% CI]
Anterior cruciate ligament	93 [83–98]		85 [62–97]		91 [82–99]		93 [83–98]		80 [56–94]		89 [80–98]		0.83 [0.70–0.97]
Medial meniscus	83 [70–93]		71 [51–87]		79 [67–91]		85 [72–94]		68 [48–84]		79 [67–91]		0.89 [0.77–0.99]
Lateral meniscus	54 [33–73]		92 [81–98]		79 [67–91]		58 [37–77]		82 [69–91]		74 [63–94]		0.74 [0.58–0.92]

*p*-value range: 0.25–1.00.

Regarding the detection of ACL tears, the overall combined sensitivity, specificity, and accuracy were similar for the two techniques. For the 2D protocol, the overall combined positive and negative predictive values were 95% and 81%, respectively. For the 3D protocol, the overall combined positive and negative predictive values were 93% and 80%, respectively.

There was no significant difference between the protocols for the detection of ACL tears. The kappa agreement between the two methods was 0.83 (range, 0.70-0.97). Regarding the detection of medial meniscal tears, the overall combined sensitivity, specificity and accuracy were similar for the two techniques. For the 2D protocol, the overall combined positive and negative predictive values were 83% and 71%, respectively. For the 3D protocol, the overall combined positive and negative predictive values were 82% and 73%, respectively.

**Figure 1:**
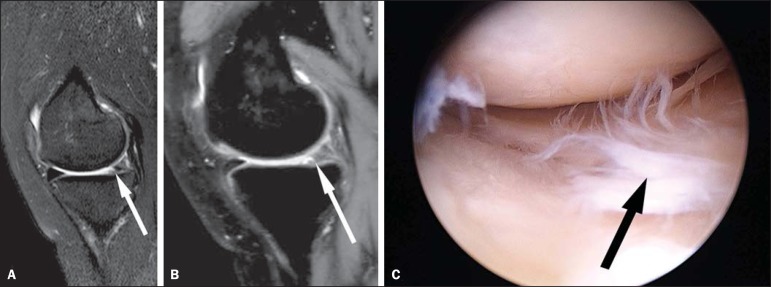
A 36-year-old woman with a tear of the medial meniscus. Sagittal MRI (**A**: 2D TSE; and **B**: 3D TSE volume isotropic turbo spin-echo acquisition [VISTA]) of the knee depicting a tear of the posterior horn of the medial meniscus (arrows). **C**: Corresponding arthroscopic correlation of the tear (arrow).

There was no significant difference between the protocols for the detection of medial meniscal tears (Figure 1). The kappa agreement between the two methods was 0.89 (range, 0.77-0.99).

Regarding the detection of lateral meniscal tears, the overall combined sensitivity, specificity and accuracy were similar for the two techniques. For the 2D protocol, the overall combined positive and negative predictive values were 78% and 79%, respectively. For the 3D protocol, the overall combined positive and negative predictive values were 63% and 79%, respectively.

There was no significant difference between the protocols for the detection of lateral meniscal tears. The kappa agreement between the two methods was 0.74 (range, 0.58-0.92).

Table 4 shows the sensitivity, specificity, accuracy, and intraobserver agreement regarding the detection of ACL, medial meniscal, and lateral meniscal tears, for both readers separately. There were no significant differences between the two protocols in terms of sensitivity, specificity, positive predictive value, negative predictive value, or accuracy, in the separate or combined readings (*p*-values ranging from 0.25 to 1.00).

**Table 4:** Sensitivity, specificity, accuracy, and intraobserver agreement (kappa) for the 2D and 3D MRI protocols in the detection of anterior cruciate ligament, medial meniscus, and lateral meniscus tears for both readers separately.

	Reader 1												
	2D						3D						
	Sensitivity		Specificity		Accuracy		Sensitivity		Specificity		Accuracy		Intraobserver agreement
MRI feature	% [95% CI]		% [95% CI]		% [95% CI]		% [95% CI]		% [95% CI]		% [95% CI]		Kappa [95% CI]
Anterior cruciate ligament	93 [77–99]		90 [56–100]		89 [74–100]		89 [72–99]		80 [44–97]		87 [71–100]		0.87 [0.70–1.00]
Medial meniscus	83 [63–95]		64 [35–87]		76 [58–95]		88 [68–97]		57 [29–82]		76 [58–95]		0.88 [0.74–1.00]
Lateral meniscus	54 [25–81]		92 [74–99]		79 [59–94]		62 [32–86]		76 [55–91]		71 [49–85]		0.70 [0.70–0.57]
	Reader 2												
	2D						3D						
	Sensitivity		Specificity		Accuracy		Sensitivity		Specificity		Accuracy		Intraobserver agreement
	% [95% CI]		% [95% CI]		% [95% CI]		% [95% CI]		% [95% CI]		% [95% CI]		Kappa [95% CI]
Anterior cruciate ligament	93 [77–99]		80 [44–97]		89 [74–100]		96 [82–100]		80 [44–97]		92 [77–100]		0.79 [0.57–0.99]
Medial meniscus	83 [63–95]		79 [49–95]		82 [62–98]		83 [63–95]		79 [49–95]		82 [62–98]		0.89 [0.75–1.00]
Lateral meniscus	54 [25–81]		92 [74–99]		79 [59–94]		54 [25–81]		88 [69–97]		76 [56–91]		0.79 [0.48–0.94]

*p*-value range: 0.25–1.00.

The interobserver agreement for the detection of ACL, medial meniscal, and lateral meniscal tears, for both MRI techniques, is presented in Table 5. There were no significant differences between the two methods in terms of the kappa interobserver agreement (*p*-values ranging from 0.58 to 0.99). On 2D and 3D TSE images, both readers correctly detected the two PCL tears that were found by the surgeons, as well as correctly identifying normal morphology of the PCL in the remaining cases.

**Table 5:** Interobserver agreement (kappa) for the 2D and 3D MRI protocols for detecting tears of the anterior cruciate ligament, medial meniscus, and lateral meniscus.

MRI feature	Interobserver agreement		
	2D TSE		3D TSE VISTA
	Kappa [95% CI]		Kappa [95% CI]
Anterior cruciate ligament	0.80 [0.61–0.99]		0.73 [0.48–0.95]
Medial meniscus	0.89 [0.75–1.00]		0.77 [0.58–0.98]
Lateral meniscus	0.56 [0.26–0.86]		0.52 [0.24–0.88]

*p*-value range: 0.58–0.99. VISTA, volume isotropic turbo spin-echo acquisition.

## DISCUSSION

Imaging methods are essential for the evaluation of knee injuries^(16,17)^. Among the various methods, MRI is one of considerable importance. Three-dimensional MRI sequences obtained with isotropic or nearly isotropic resolution techniques can be manipulated to provide high-resolution multiplanar reconstructions.

The diagnostic performance of several 3D isotropic-type gradient-echo sequences has been previously tested in the evaluation of articular cartilage pathology^(18-20)^: spoiled gradient-recalled echo; double-echo steady-state; driven equilibrium Fourier transform; fast low-angle shot; and balanced steady-state free precession.

However, 3D gradient-echo acquisition protocols are time consuming and cannot completely replace routine 2D TSE, because they do not allow accurate assessment of other important joint structures such as the menisci, ligaments and subchondral bone changes^(18-20)^.

Recently, 3D TSE MRI techniques were introduced that provide isotropic or nearly isotropic resolution. Previous studies have shown that this technique has good diagnostic performance for the detection of cartilaginous, meniscal, and ligament lesions with a 3.0 T magnet^(1,4-10)^. It should also be noted that acquisition of the source images was significantly faster with the 3D TSE protocol than with the triplanar 2D TSE protocol.

The volume isotropic turbo spin-echo acquisition MRI technique provides high-resolution volumetric intermediate-weighted images acquired with 3D TSE and is clinically available for 1.5 and 3.0 T systems. To our knowledge, there have been no previous studies testing the diagnostic performance of this technique in detecting meniscal and ligament tears of the knee in a 1.5 T scanner and comparing the results promptly with arthroscopic correlation.

We found that there were no significant differences between 2D and 3D TSE techniques regarding the detection of ACL and meniscal tears, with substantial to almost perfect intraobserver and interobserver agreement for medial meniscal and ACL tears. Similar results have been obtained in previous studies testing 3D TSE sequences^(1,4-7)^.

The only discrepancy between our study and those in the current literature was the relatively lower sensitivity for lateral meniscal tears in both MRI techniques, with moderate interobserver agreement^(1,4-10)^. We retrospectively reviewed the six cases for which lateral meniscal tears were missed on MRI and found that four of them were very small peripheral flap tears in patients with complete ACL tears or extensive medial meniscal tears. The fact that those tears were subtle even in arthroscopy probably influenced our results. However, the purpose of the present study was to compare the diagnostic performance of the two techniques.

Two previous studies presented different from ours results when testing the 3D TSE sequence in the knee joint and concluded that 2D TSE acquisitions are more reliable than are 3D TSE acquisitions^(8,10)^. Van Dyck et al. suggested that, although 3D TSE may be a valuable component of a knee MRI protocol at 3.0 T, it cannot entirely replace routine 2D MRI in the assessment of the knee^(10)^. However, other authors have tested 3D TSE MRI of the knee and concluded that its diagnostic performance is comparable to that of conventional 2D sequences in the detection of meniscal and ACL tears^(6,7)^. Ai et al. stated that the 3D TSE sequence is comparable if not superior to conventional 2D imaging for comprehensive joint assessment of knee injuries and predicted that it is likely to replace the currently used 2D imaging protocols for the evaluation of knee injuries^(7)^.

This study has some notable strengths. We had the opportunity to optimize the time between MRI acquisition and arthroscopy, which were performed on the same day in 80% of the cases, with a maximum interval of three days. In previous studies, the mean time between imaging and arthroscopy has ranged from four weeks to four months, which could compromise the reliability of the imaging findings in relation to the surgical data^(1,4-10)^. In addition, the different experience levels of the readers in our study did not appear to have a significant effect on the final diagnostic performance, which suggests that the 3D TSE technique can also be successfully interpreted by readers with varying degrees of expertise.

Our study has some limitations. We had a relatively small sample (38 subjects), which made it impossible to analyze the diagnostic performance of 3D TSE for PCL tears, because there were only two positive cases. In addition, we did not evaluate the articular cartilage. The complexity of grading cartilage pathology and the potential assessment of cartilage pathology in different regions/compartments of the knee using the 3D TSE technique would require careful standardization, as well as the evaluation of a much larger sample, in order to achieve adequate statistical power. Furthermore, the relatively thick slices used for the standard 2D TSE sequences (4.0 mm) could potentially reduce the sensitivity and specificity of that technique. Moreover, there was an inherent referral bias in that only patients referred for arthroscopy were included in the study. There was also a verification bias, because the readers knew which MRI technique they were reading (separate rolls of readings for each protocol and obvious imaging features). The minimum interval of four weeks between the two readings was intended to minimize interpretation and recognition bias. Another potential limitation was that we did not categorize in detail the types and locations of the meniscal and cruciate ligament tears. With such a small sample size, we would not have had enough statistical power to test the ability of the two techniques to depict different types and locations of tears, in comparison with that of arthroscopy.

Although the readers had more previous experience with the 2D TSE sequences than with the 3D TSE technique, the novelty was reduced because they had been assessing 3D TSE images in clinical practice for at least 12 months prior to the study, as recommended in the literature^(8)^. On the basis of the results of the present study, we cannot immediately recommend that radiologists replace the routine 2D TSE technique with the 3D TSE protocol for knee MRI, because our assessment was limited to the menisci and cruciate ligaments. However, we feel that the results of this study represent an important step toward implementing this technique in clinical practice.

In conclusion, we demonstrated that 3D TSE is a reliable technique and has a diagnostic performance similar to that of the routine 2D TSE MR protocol for detecting meniscal and ACL tears at 1.5 T. The 3D TSE MRI technique has the advantage of faster acquisition times, which would be important in clinical practice to increase patient comfort and the efficiency of the MRI scanner.
